# Immunosenescence in aging: between immune cells depletion and cytokines up-regulation

**DOI:** 10.1186/s12948-017-0077-0

**Published:** 2017-12-14

**Authors:** Maria Teresa Ventura, Marco Casciaro, Sebastiano Gangemi, Rosalba Buquicchio

**Affiliations:** 1 Department of Interdisciplinary Medicine, University of Bari, Policlinico, Piazza G. Cesare no 11, 70124 Bari, Italy; 2 School and Operative Unit of Allergy and Clinical Immunology, Department of Clinical and Experimental Medicine, University of Messina, Messina, Italy; 3 Dermatological Clinic, Department of Biomedical Science and Human Oncology, University of Bari Medical School, Policlinico, Italy

## Abstract

**Background:**

The immunosenescence is a relatively recent chapter, correlated with the linear extension of the average life began in the nineteenth century and still in progress. The most important feature of immunosenescence is the accumulation in the “immunological space” of memory and effector cells as a result of the stimulation caused by repeated clinical and subclinical infections and by continuous exposure to antigens (inhalant allergens, food, etc.). This state of chronic inflammation that characterizes senescence has a significant impact on survival and fragility. In fact, the condition of frail elderly occurs less frequently in situations characterized by poor contact with viral infections and parasitic diseases. Furthermore the immunosenescence is characterized by a particular “remodelling” of the immune system, induced by oxidative stress. Apoptosis plays a central role in old age, a period in which the ability of apoptosis can change. The remodelling of apoptosis, together with the Inflammaging and the up-regulation of the immune response with the consequent secretion of pro-inflammatory lymphokines represents the major determinant of the rate of aging and longevity, as well as of the most common diseases related with age and with tumors. Other changes occur in the innate immunity, the first line of defence providing rapid, but unspecific and incomplete protection, consisting mostly of monocytes, natural killer cells and dendritic cells, acting up to the establishment of a adaptive immune response, which is slower, but highly specific, which cellular substrate consists of T and B lymphocytes. The markers of “Inflammaging” in adaptive immunity in centenarians are characterized by a decrease in T cells “naive.” The reduction of CD8 virgins may be related to the risk of morbidity and death, as well as the combination of the increase of CD8+ cells and reduction of CD4+ T cells and the reduction of CD19+ B cells. The immune function of the elderly is weakened to due to the exhaustion of T cell-virgin (CD95−), which are replaced with the clonal expansion of CD28-T cells.

**Conclusions:**

The increase of pro-inflammatory cytokines is associated with dementia, Parkinson’s disease, atherosclerosis, diabetes type 2, sarcopenia and a high risk of morbidity and mortality. A correct modulation of immune responses and apoptotic phenomena can be useful to reduce age-related degenerative diseases, as well as inflammatory and neoplastic diseases.

## Background

Recent researches stigmatize that the steady increase in life expectancy in Europe, USA, Canada, Japan and England will allow many of the children born in 2000 to reach 100 years of life [1]. In this perspective, the main objective of the geriatrician is to analyze risk factors for diseases and conditions that can lead to functional limitations for the elderly, in order to avoid people to reach a disability state. In summary, the increase in life expectancy must coincide with an expectation of health, good health and self-sufficiency for the last part of life [2]. Old age is a situation in which a number of factors (molecular, cellular, physiological, immunological and psycho-social events) help to set up a scenario of “exhaustion of reserves”; this consist of a inability to functional adaptations and an accumulation of deficits of many organs [3]. This situation undergoes a dynamic process that oscillates between a “successful” and pathological aging, which establishes a situation of vulnerability that is identified with the fragility state. Undoubtedly, to the fragility state contribute the same factors that have contributed to the increased life expectancy, which is attested at the moment at 80 years of age [4]. Many factors have allowed this “stretching”, i.e. the decrease of infant mortality, antibiotic therapy and prevention of cardiovascular and metabolic diseases, but also, especially in industrialized countries, the improvement of hygienic and nutrition conditions [5]. However, if aging is not accompanied by a healthy condition, the costs related to disabilities or frailty age-related could lead to the overgrowth of the public health expense with a negative impact on social welfare.

## Immunosenescence

“The aging phenotype”, including the immunosenescence is the result of an imbalance between inflammatory and anti-inflammatory mechanisms with the consequence of a state defined by some authors as “inflammaging” [6–8]. The “Inflammaging” is due to chronic antigen stimulation that occurs in the course of life and to the oxidative stress that involves the production of oxygen free radicals and toxic products. Both these factors are able to modify the potential of apoptotic lymphocytes. In fact, the phenomenon of “remodelling” and the “up-regulation” of pro-inflammatory cytokines (including IL-6) are the components most heavily implicated in the processes of longevity and diseases related to senescence [9–12]. As pro-inflammatory and anti-inflammatory chemokines and other signalling molecules, might propagate from already activated cells to adjacent ones and systemically by circulating products and microvesicles, recent studies claimed the possibility of understanding molecular basis of inflammaging by novel omic approaches [13].

In this sense, the state of good health in the elderly is the result not only of low pro inflammatory mechanisms, but also of an efficient network capable of neutralizing anti inflammatory antigenic insults received in the course of life. For this reason, the inflammaging would not only be important for the mechanisms of immunosenescence but also for the problem of longevity. It is reported that fragility is the result of an inflammatory state associated with the overproduction of certain lymphokines, including IL-6, called cytokine of geriatricians [14]. This factor, together with hormonal changes, nutritional deficiencies and physical inactivity would lead to one of the most important component of fragility that is sarcopenia [15, 16], as well as the reduction of bone mass. In this context, immunity appears to play an important role, both in the regulation of the mechanisms of aging as well as in the onset of the diseases typical of aging (i.e. infectious diseases, autoimmunity, cancer, metabolic diseases and neurodegenerative diseases).

## Oxidative damage

Another factor influencing the Immune System, in addition to the antigenic stimulus, is the intervention of metabolites of oxygen (ROS), a consequence of the activation of the respiratory burst; as they can cause significant damage, is plausible to assume that aging is also due to an accumulation of free radicals [17]. The increase in oxygen metabolites and their accumulation causes damage to important cellular components (lipid membranes, the structural and enzymatic proteins and nucleic acids), which are contrasted by enzymatic and non-enzymatic defence systems, reparative enzymes, DNA damage and apoptotic processes damage-induced [18, 19]. These protective mechanisms become less effective as a result of continued exposure to oxidative stress and the accumulation of senescent and mutated cells, leading to an increased risk of cancer. The p53 protein counteracts the development of a neoplasm flaunting how cells respond to injury (DNA repair, or, if it fails, apoptosis). p53 plays an important role in senescence: if it increases, the incidence of neoplasia is reduced, but on the other hand it increases the speed of aging [20]. The result is a delicate balance between reduced p53 that leads to death by cancer and increased p53 leading to death for acceleration of senescence. Furthermore, an over expression of p53 generates reactive oxygen intermediates [21].

An important source of oxygen intermediates are the mitochondria. Although the main mitochondrial function is the production of energy, the isolated mitochondria generate oxygen radicals during oxidative phosphorylation. The mitochondrial electron transport chain is imperfect because it generates a superoxide radical by a process of reduction of O_2_. The enzymatic dismutation of the superoxide radical produces H_2_O_2_, another important biological oxidant. Another factor contributing to the senescence is apoptosis, especially the one induced by acid metabolites arising from the paths of lipoxygenase and cyclooxygenase; however it is not clear whether these products induce the production of reactive intermediates or if they act independently as oxidants to induce apoptosis. Another intermediate product of oxygen metabolism is nitric oxide, a free radical known as an important regulator of mitochondrial function, capable of increasing the apoptotic phenomena, but also, at physiological levels, of preventing apoptosis and of interfering with the cascade of capsaicin [22]. In conclusion, high levels of oxidants can change the potential of oxido-reduction, by reducing the levels of ATP and increasing the porosity of the membranes leading to a progressive aging phenomenon of the cells, included the Immune System ones [23].

## The “Remodelling” of the immune system

As a result of ROS accumulation, cells become resistant to apoptosis -induced damage and the number of senescent cells increases, while chronic antigenic stimulation induces increase of activated immune cells and overproduction of pro-inflammatory lymphokines that contribute to the remodelling of the immune system and of the ‘inflammaging’. Senescence is a highly dynamic phenomenon characterised by continuous body adaptation to deteriorative changes [24].

ROS are closely linked to senescence and age-related diseases, in fact, genomic instability, caused by oxidative damage is the primary cause of aging. A caloric restriction can increase the average life-attenuating oxidative stress caused by normal metabolism [25].

As result of both inflammaging and of ROS increase, the modulation of apoptosis mechanisms becomes particularly delicate during senescence. The reduced sensitivity to the damage-induced apoptosis, typical of senescent cells, contributes to the accumulation of dysfunctional cells, clones of CD8+ and memory cells with a reduction of immunological space and an increased risk of infections and neoplastic diseases or degenerative disorders. The increase of the activation-induced apoptosis in response to inflammatory cytokines contributes to: the depletion of cells “naive”, the reduction of the capacity of clonal expansion, the reduction of T cell responses with decreased ability to mount strong immune responses to antigenic stimuli and reduction of the immune repertoire.

The shortening of the telomeric DNA is age specific, and, regardless of the genetic influence, it is the result of the immunological history of each individual, with a close association between telomere length and mortality of individuals older than 65 years [26]. The input of virgin T cells gradually decreases, and, recently, the marker of the lymphocytes of the new generation are the T REC which represent markers of replication of T cells, with a progressive reduction of each subsequent division. Of course, the T REC decay dramatically with age in peripheral T lymphocytes.

## Apoptosis

Apoptosis, a complex mechanism of programmed cell death, allows the maintenance of a physiological homeostasis mechanisms between survival and removal of damaged cells, allowing also the prevention of many diseases including neoplastic ones. Apoptosis is a strategic mechanism for the manifestation of the clonotypic diversity during lymphocyte selection, permitting to control the clonal expansion after antigenic stimulation. Apoptosis can be induced after a cellular damage (damage-induced cell death), or can be “activated” by a series of signals and anchor ligands to programmed-death receptors (activation-induced cell death). Apoptosis is part of many changes typical of the immunosenescence, such as thymic involution, the alteration of the “repertoire” of T cells and the accumulation of effector memory cells, all events at the basis of autoimmunity. Studies about apoptosis in aging are controversial. In fact, during senescence both the two apoptotic process can be modulated differently, resulting in a variable impact in the process of senescence. A proper modulation of this important function can extend the lifespan and reduce the degenerative processes and inflammatory and neoplastic diseases that are very common during senescence [6, 27].

## Hematopoietic bone and thymus

The immune system cells are constantly renewed from hematopoietic stem cells (HSC), but this ability declines during senescence and the total amount of hematopoietic tissue decreases. This event also seems to correlate with telomere shortening [28]. The changes affect also the myeloid and erythroid progenitors as B cells, with the consequence of a reduction in mature B cells. The precursors of T cells seem to suffer less; the changes that occur with age to the thymus gland, also lead to changes in the T cell compartment. The thymus undergoes a process of physiological involution, with volume reduction and replacement with adipose tissue in the functional part of both cortex and medulla, contraction of soluble factors and hormonal cytokines production. This process begins early in life and is almost complete at the age of 40–50 years [29].

Moreover, the immune system has the important function to protect the body from any form of damaging agent (chemical, traumatic or infectious). There are two kind of immunity working together in a cooperative manner: the natural immunity (innate) immunity and adaptive (acquired). The natural immunity is the first line of defence because it provides a fast protection, but unspecific and incomplete, consisting mostly of monocytes, natural killer cells and dendritic cells, which acts until the adaptive immune response is established; this immune response is slower, but highly specific and permanent, with a cellular substrate consisting of T and B lymphocytes.

### T cells

The T cells are generated through a Thymic selection and they can be distinguished in CD4+ and CD8+, by their co-receptor molecules. These two cellular subtypes show during the aging process of the organism some changes in their percentages: the CD8+ cells increase their number during senescence. The CD4+ and the CD8+ cells express mutually exclusive the phenotype CD45RA and CD45RO. The first phenotype makes let to recognize naïve T cells, instead the second one to individuate memory/activated T cells [30]. The reduction of naïve lymphocytes may be a consequence of both thymic involution and chronic antigenic stimulation [31]. This event helps to explain the reduced ability of the elderly to resist to new infections [32].

Furthermore in the elderly naïve T-cells show multiple alterations, including the shortening of telomeres, the reduced production of IL-2 and the diminished ability to differentiate themselves into effector-cells. The loss in the number and function of the naïve T-cells is compensated in about 30% of the elderly, with the expansion of T CD8+ , CD45RO+, CD25+ clones, capable of producing IL-2, and with a protective humoral capacity towards vaccinations with the expansion of effector “memory” cells [33]. In particular, in the elderly the vaccinations induce the accumulation of CD8+ effector cells with phenotypic changes, such as the loss of costimulatory CD8 molecules [32].

CD28-cells are responsible for the production of proinflammatory cytokines and are resistant to apoptosis. The origin of the CD28-cells has not yet been fully elucidated, but it is assumed that they represent cells undergoing a replicative senescence, due to the shortening of telomeres and to a reduction of the proliferative capacity [34]. The inversion of the CD4+/CD8+ cell number ratio, the increased number of the memory-effector cells and the seropositivity for the Citomegalvirus (CMV), identify an immune risk phenotype (IRP) in elderly patients [35]. At the same time in elderly patients it has been shown an increased production of IL-1, IL-4, IL-6 and IFN-gamma. These cytokines control B Cells differentiation through the isotype switch and the Ig production.

Further alterations concern a compromised response to the oxidative stress, that causes an increased susceptibility to damage-induced cell death [36], and calcium flow kinetics [37]. Recently it has been associated with the senescence a reduction of Mir 181 (MicroRNA precursor), that in T cell causes an impairment in the antigen recognition [38].

Regulatory T cells (Tregs) are a subset characterized by a high expression of CD25 and FOXP3, a transcriptional factor for the function and differentiation of Treg cells. The number of CD4+ FOXP3+ lymphocytes increases in the senile age. The accumulation of these cells in the elderly plays an important role in reactivating chronic infections and the change in the T17/Treg ratio can cause alterations in immune response with the appearance of inflammatory or autoimmune diseases [39].

### B cells

Also the reservoir of B cells is influenced by age. In fact, humoral immunity undergoes both quantitative and qualitative alterations [40].

The reduced function of B cells was thought being due to a lack of helper T function in T-dependent responses. On the other hand, there are functions of B cells which are T-independent; one example is the response to the polysaccharide, which is crucial for antibacterial protection and that seems to be inefficient too [41].

In addition, some data suggest that B cells are important antigen-presenting cells themselves and that can be regulatory with key function for the development of T cells. Therefore, it is conceivable that some of the lack in the T cell functions may be due to an insufficient help from B cells. At the same time, changes are described in the number of B cells. In the elderly, there are also reported reduced levels of IgM and IgD (M and D type immunoglobulins) certainly connected to the transition from naive cells to memory B cells area [42]. On the contrary, during the senescence it occurs an increase of the IgG (G type immunoglobulins) level, especially of IgG1, IgG2 and IgG3; the level of IgA is also increased [43]. In particular, IgAs undergo significant changes, with a marked increase of monomeric IgA1, both in serum and saliva and a reduction of polymeric IgA2, especially in the sputum [44]. This imbalance could be charged to the reduction of the Peyer’s patches at the level of the gastrointestinal mucosa as regards as IgA2, while the increase of IgA1 may be secondary to a deficiency of the activity of T “suppressor” subset and consequent hyperfunction of B lymphocytes [45]. The deficits taking place in this area are largely due to infectious events in the elderly, particularly in the gastrointestinal and respiratory system. The reduced number of plasma cells in the elderly bone marrow [46] causes a lack of antibody production, a reduced ability to respond to viruses and bacteria [47] and an altered response to vaccines against B hepatitis virus [48].

## The innate immunity system

Alterations in innate immunity have a crucial role and the amount of related studies have identified a trend in the chapter of immunogerontology starting with the reduction of barriers in the epithelial layer of the skin and gastrointestinal and respiratory mucosa [49] with a consequent changes in local immunoglobulin ratio. Moreover, even some physiological events, such as the reduction of the thymic mass, seems to support the hypothesis that the immune system plays an important role in the phenomenon of aging, thus justifying a theory to explain some of the immunological diseases typical of this age such as autoimmune diseases, malignancies and infections. The high incidence of infectious events in old age can, however, be secondary to alterations in the phagocyte system [50].

With regard to the skin, immunosenescence is characterized by an impairment of all the structures with loss of the “barrier” function, reduction of the number and the volume of hairs, reduction of the number of sebaceous glands, loss of skin elasticity, impairment of the immunological defence of the skin [51].

Dendritic cells, responsible for the very first recognition of pathogens in the skin, show mitochondrial dysfunctions that interfere with their protective role [52].

In particular there is an impairment of the antigen uptake and of the apoptotic function [53].

Comparing the elderly plasmocytoid dendritic cell (PCD) capacity of antigen uptake with the one of the PCD of the young it is possible to observe a reduced ability of the elderly PCD to induce proliferation and stimulate secretion of INF gamma in CD4+ and CD8+ cells [54].

### Macrophages

Macrophages, able to produce pro-inflammatory cytokines (TNF-alpha, IL-1, IL-6 e IL-8), have the function of processing and presenting antigens to T cells. During senescence, a decrease of macrophages precursors has been described, instead the number of monocytes appears unchanged [55]. The shortening of telomeres occurring in the senile age results in a reduction in the production of GS-CSF but also of Cytokines such as TNF-alpha and IL-6 [56]. In older animals it occurs a reduction of the production of superoxide anion after incubation with INF-gamma [57].

The phagocytic function appears to be reduced, while, chemotaxis seems to be conserved, especially in the presence of certain factors such as stimulants of the complement fragment C5a. The production of lymphocyte derived chemotactic factors (LDCF) is reduced, as well as chemotaxis in the presence of this stimulator factor. In this case the inhibitory mechanism appears to be related to prostaglandins that are produced in high quantities, during senescence, and which exert an inhibitory action [58]. The reduced production of LDCF could be related to a low percentage of lymphocytes involved in the synthesis of the cytokine.

### Neutrophils

Their number is preserved in the elderly, while the expression of CD16 Fc gamma receptor is reduced, with the consequence that, both the generation of superoxide mediated by the Fc receptor and the phagocytosis are impaired in the elderly; this suggests that the decline of the effector response of Fc receptors is particularly important for neutrophil dysfunction of the elderly [59].

In elderly people, the reduced response of these cells to Streptococcus Aureus is of fundamental clinical importance, because this event increases susceptibility to lung infections. At the same time in the aged mice the migration of neutrophils into the lungs is reduced and this increases the risk of pulmonary infections and recurrences [60].

In addition, very recently, an alteration of the pathogen-mediated destruction of neutrophil extracellular Traps (NETs) has been described, confirming the reasons of the increase of infections in the elderly [61].

### NK cells

The high incidence of immunoproliferative diseases in the elderly suggests that in this age a deficiency of an important mechanism of immune surveillance such as NK activity can be occur. In 1986, through the use of a “slow” target such as a cell line derived from an hepatocellular carcinoma, allowing an optimal evaluation of NK function, it was conceivable to demonstrate that in the elderly it was a significant reduction of the spontaneous cytotoxic capacity [62]. Recent studies pointed out that a high NK cytotoxicity is associated with longevity and good health, while a low NK function is associated with an increase in morbidity and mortality, and consequently, infections, mechanisms of atherosclerotic and neurodegenerative diseases. Furthermore, NK cells by producing cell lysis could cause the release of perforin and granzymes, which, in turn, activate caspases and provoke apoptosis of target cells. During senescence it occurs the reduction of an important lymphokine for the lymphocyte activation processes like the IL-2 and also for the killing of the NK-resistant cell lines in response to IL-2. This contributes to the deficit of the function, even in the presence of a normal number of NK cells [63].

Particularly during senescence there is a redistribution of NK cells with decreasing CD56 cells, characterized by a high density of surface CD56 antigen. In contrast, there is an increase in CD56–CD16 Nk cells [64]. This results in a reduction in IFN secretion for the elderly compared with the secreted quantity in young subjects [65]. In addition, during the senescence there is a decrease in the expression of the receptor activation expression, especially linked to the receptor NKp30 and NKp46 [66].

Of course, it’s easy to imagine the consequences that may follow an alteration to the function of this population during senescence; in fact, NK cells intervene both in the elimination of tumor or viral-infected cells and also in the innate and adaptive immunological regulation, through the production of cytokines and chemokines [67].

## Phenotype of immunological RISK (IRP) during senescence

According to recent studies, the IRP is predictive of the development of cognitive deficits and, as outlined by some authors, [35] is a prelude for a mortality rate over the next 4 years in 58% of cases. The IRP is defined, in the studies of this group of Swedish researchers carried out on elderly octogenarians and nonagenarians, by identifying some characteristic “markers” of this phenotype, including the inversion of the CD4/CD8 ratio, the increase in CD8 * CD28-memory/effector cells, the increase of proinflammatory cytokines such as IL-6, the reduction of B lymphocytes and a marked seropositivity for cytomegalovirus [68].

The pro-inflammatory profile resulting from an interaction between the genotype and environmental factors, becomes strategic trough the years; the increase of cytokine secretion also correlated with the impact of cytomegalovirus infection is responsible for an unsuccessful aging [69]. CMV-specific cells, both CD4+ and CD8+ cells have short telomeres and this leads to chromosomal instability and DNA damage repair processes in growth arrest and/or apoptosis. The consequence is that not all T memory cells differ in the same way and that can happen an expansion of this cell pool, whose clinical consequences consist in an increase in infectious diseases and neoplasms [70].

## Conclusion

A shown above, immunosenescence is an unavoidable process typical of life being. Many immune system cells undergoes this process; however, the senescence process differ from one subject to the other. The development of a pro-inflammatory cytokines phenotype together with the counterbalance of an anti-inflammatory profile could let people reach an old age without disability. A correct modulation of immune responses and of apoptotic phenomena, in fact, can be useful to reduce age-related degenerative diseases, as well as inflammatory and neoplastic diseases in order to reach a successful aging.
